# Photosynthetic bacterium *Rhodopseudomonas palustris* GJ‐22 induces systemic resistance against viruses

**DOI:** 10.1111/1751-7915.12704

**Published:** 2017-03-14

**Authors:** Pin Su, Xinqiu Tan, Chenggang Li, Deyong Zhang, Ju'e Cheng, Songbai Zhang, Xuguo Zhou, Qingpin Yan, Jing Peng, Zhuo Zhang, Yong Liu, Xiangyang Lu

**Affiliations:** ^1^ Hunan Academy of Agricultural SciencesHunan Plant Protection InstituteChangsha410125China; ^2^ Department of EntomologyUniversity of KentuckyLexingtonKY40546USA; ^3^ College of Bioscience and BiotechnologyHunan Agricultural UniversityChangsha410128China

## Abstract

Photosynthetic bacteria (PSB) have been extensively used in agriculture to promote plant growth and to improve crop quality. Their potential application in plant disease management, however, is largely overlooked. In this study, the PSB strain *Rhodopseudomonas palustris* GJ‐22 was investigated for its ability to induce resistance against a plant virus while promoting plant growth. In the field, a foliar spray of GJ‐22 suspension protected tobacco plants against tobacco mosaic virus (TMV). Under axenic conditions, GJ‐22 colonized the plant phyllosphere and induced resistance against TMV. Additionally, GJ‐22 produced two phytohormones, indole‐3‐acetic acid and 5‐aminolevulinic acid, which promote growth and germination in tobacco. Furthermore, GJ‐22‐inoculated plants elevated their immune response under subsequent TMV infection. This research may give rise to a novel biological agent with a dual function in disease management while promoting plant growth.

## Introduction

Disease resistance can be induced and enhanced by previous exposure to biotic or abiotic stimulus (Conrath *et al*., 2006). This process is called priming, and primed plants systemically initiate rapid and robust defence responses upon future challenges (Pastor *et al*., 2013). Based on the nature of the eliciting agents and the subsequent biochemical responses, induced resistance is categorized into systemic acquired resistance (SAR) and induced systemic resistance (ISR). Induced by prior necrotrophic pathogen attack, SAR depends on the salicylic acid (SA)‐inducible defence mechanism (Vallad and Goodman, 2004). In contrast, ISR is induced by the non‐pathogenic microorganisms and depends on the jasmonic acid (JA)/ethylene (ET)‐inducible defence mechanism (Derksen *et al*., 2013). In practical applications, the establishment of SAR is usually mimicked by the exogenous application of synthetic chemicals, such as the functional analogues of salicylate, e.g. benzo (1,2,3) thiadiazole‐7‐carbothioic acid S‐methyl ester (BTH) (Vallad and Goodman, 2004). The onset of ISR is generally achieved by the employment of plant growth‐promoting bacteria (PGPB) and more commonly by plant growth‐promoting rhizobacteria (PGPR), which account for the absolute majority of the extensively studied ISR‐inducing strains (Van Loon, 2007). The integration of ISR induction and growth promotion is considered to be a promising avenue for overcoming the fitness cost imposed by chemical inducers (Owen *et al*., 2014). The superiority of ISR‐eliciting PGPB has been notably manifested during glasshouse tests and field trials and has increasingly motivated candidate‐strain selection efforts on a worldwide scale (Wang *et al*., 2014; Berg, 2015). A large spectra of candidate strains have been isolated and characterized for their inducing capacities and amplitudes of protection, but as documented, most of the strains have converged into merely a few families, such as *Bacillus* spp. (Murphy *et al*., 2000)., *Pseudomonas* spp. (Maurhofer *et al*., 1994) and *Serratia* spp. (Raupach *et al*., 1996), among others (Vallad and Goodman, 2004).

While extensive research of ISR‐eliciting strains is being conducted among PGPR strains and endophytic bacteria, the potential of photosynthetic bacteria (PSB) has been largely overlooked. A phylogenetically diverse group, PSB, is well known for their wide distribution in water ways and their metabolic versatility, which includes nitrogen and carbon dioxide fixation and desulfurization (Sasikala and Ramana, 1998). Photosynthetic bacteria have been used extensively in agricultural production to promote plant growth and to improve crop quality (Koh and Song, 2007). *R. palustris* is a typical purple non‐sulfur photosynthetic bacterium. It produces an array of chemicals, including siderophores, riboflavin, 5‐aminolevulinic acid (ALA), extracellular polymeric substances (EPS) and bacterial acyl‐homoserine lactone (AHL) (Sasaki *et al*., 2005), which can trigger ISR in plants (Pieterse, 2009). The wealth of these chemicals in PSB metabolites leads to the speculation that PSB strains may also possess ISR‐inducing properties.

Built on this knowledge, we hypothesized that the application of PSB can boost plant immune responses. To examine this hypothesis, we screened our PSB culture collection against tobacco mosaic virus (TMV) on tobacco plants. GJ‐22, an isolate with a cypermethrin degradation ability that was previously characterized to be *R. palustris* (Yin *et al*., 2012), exhibited efficacious control against TMV infection. This result was also verified through field trials. Not surprisingly, under axenic conditions, GJ‐22 significantly improved plant growth and seed germination in tobacco plants. This is, at least partially, attributed to the production of IAA and ALA. Moreover, GJ‐22‐inoculated tobacco seedlings were resistant to TMV infection. To explore the full potential of photosynthetic bacteria in agricultural practices, this research provides the empirical evidences for the future development of a novel biological agent with a dual function in promoting plant growth while controlling pathogenic attacks.

## Results

### Plant growth and seed germination promotion under axenic conditions

**Figure 1:**
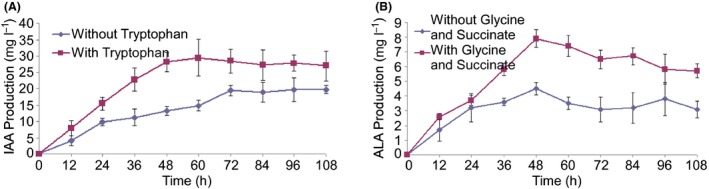
Production of IAA and ALA by *R. palustris* GJ‐22. A. IAA production from two sets of cultures supplemented with and without the precursor tryptophan. B. ALA production from two sets of cultures supplemented with and without the precursors glycine and succinate. The values are the mean of the detected concentrations with the standard deviation based on 12 cultures in a representative experiment that was repeated four times with similar results.

GJ‐22 exhibited the synthesis of both chemicals indole‐3‐acetic acid (IAA) and 5‐aminolevulinic acid (ALA). Under anaerobic conditions, the highest concentration of IAA reached 29.5 mg l^−1^ in liquid culture 60 h after inoculation when the IAA precursor tryptophan (3 mM) was added to the culture medium, significantly higher than 14.8 mg l^−1^ when IAA precursor was not added, and the concentration remained insignificantly different at the tested times afterwards, indicating a consistent capability for IAA synthesis (Fig. 1A). The ALA concentration dynamic of GJ‐22 in liquid culture appeared slightly different from that of IAA (Fig. 1B). The highest concentration reached 7.9 mg l^−1^ at 48 h after inoculation when the ALA precursors glycine and succinate (15 mM) were added to the culture medium, significantly higher than 4.5 mg l^−1^ when ALA precursors were not added. Subsequently, these levels reduced to 5.7 mg l^−1^ at 108 h after inoculation. When GJ‐22 was cultured without precursor supplements in the culture medium, lower IAA and ALA productions at all testing times were detected compared with the precursor supplement conditions.

**Figure 2:**
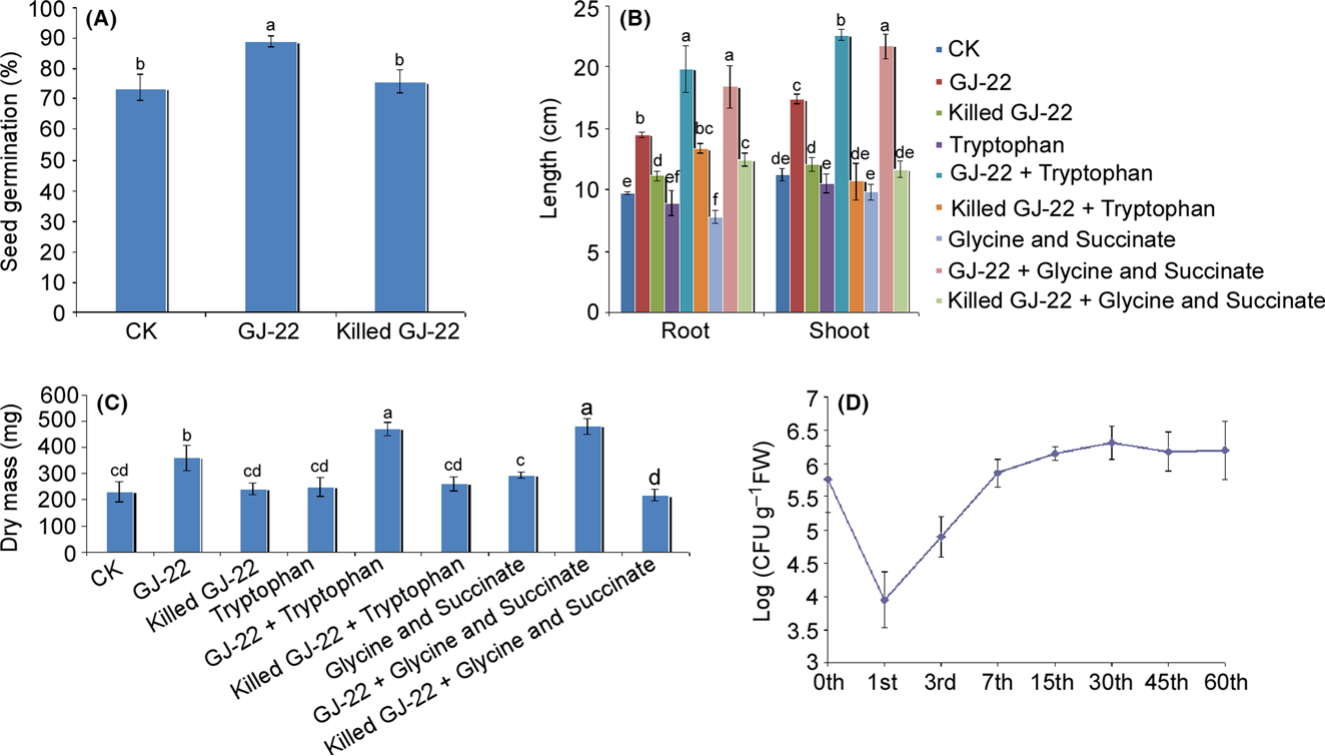
Seed germination and growth promotion effect of the phyllospheric colonization of *R. palustris* GJ‐22. A. The seed germination rates of three treatments were calculated based on 100 seeds 7 days after treatment. CK was ultrapure water. B. The root and shoot lengths of nine treatments were calculated 21 days after treatment. C. The dry mass of each whole plant was assessed after drying at 80°C for 7 days. D. The phyllospheric colonization of the intrinsically antibiotic‐resistant (kanamycin and cycloheximide) strain GJ‐22‐1 was detected based on one gram of fresh leaf at different time points. The root and shoot length and dry mass data are expressed as the mean values with the standard deviations based on 100 plants in a representative experiment that was repeated four times with similar results. The seed germination rate and phyllospheric colonization data are expressed as the mean values with the standard deviations from four replicates. The different letters within the same data group indicate significant differences between the treatments as determined by Fisher's LSD (*P* = 0.05).

The germination of tobacco seeds under axenic conditions was promoted by GJ‐22 treatment (Fig. 2A). The seed germination rate in the GJ‐22 treatment was 88.9%, which was significantly higher than the rates following treatment with ultrapure water and killed GJ‐22, which were 73.8% and 75.8% respectively. No significant difference was found between the ultrapure water and killed GJ‐22 treatment conditions.

The root and shoot lengths (Fig. 2B) and dry masses (Fig. 2C) of the plants treated with the GJ‐22 suspension and the chemical‐supplemented GJ‐22 suspension were significantly higher than those of the plants treated with ultrapure water, the killed GJ‐22 suspension and the chemical‐supplemented killed GJ‐22 suspension. The GJ‐22 suspension supplemented with both phytohormone precursors elicited significantly greater growth promotion effects than the GJ‐22 suspension alone. The root and shoot length increased 49.5% and 55.4% by GJ‐22 suspension, compared with water control. But when GJ‐22 suspension was supplemented with chemical precursors, greater root and shoot length increases were resulted compared with water control, which were 104.1% and 101.8% for IAA precursor, and 89.6% and 93.8% for ALA precursors. The same pattern of increases was also observed in terms of dry mass weight. The precursor‐supplemented GJ‐22 suspension resulted in a greater increase in dry mass than did the GJ‐22 suspension treatment alone.

### Phyllospheric colonization of *R. palustris* GJ‐22

The phyllospheric colonization of GJ‐22 was also detected under axenic conditions (Fig. 2D). The GJ‐22 suspension was applied to tobacco seedlings as a foliar spray with an inoculation concentration of 6 × 10^7^ CFU ml^−1^. Within 24 h, the detected foliar population of fresh leaves dropped drastically from 5.8 × 10^5^ CFU g^−1^ to 0.9 × 10^4^ CFU g^−1^. Subsequently, progressive restoration of the population was detected within 7 days, and the concentration reached 7.1 × 10^5^ CFU g^−1^ on the 7th day. The population size remained stable afterwards, and a concentration of 1.6 × 10^6^ CFU g^−1^ was detected on the 60th day after inoculation.

### Post‐challenge immune response assays

#### TMV accumulation assay

**Figure 3:**
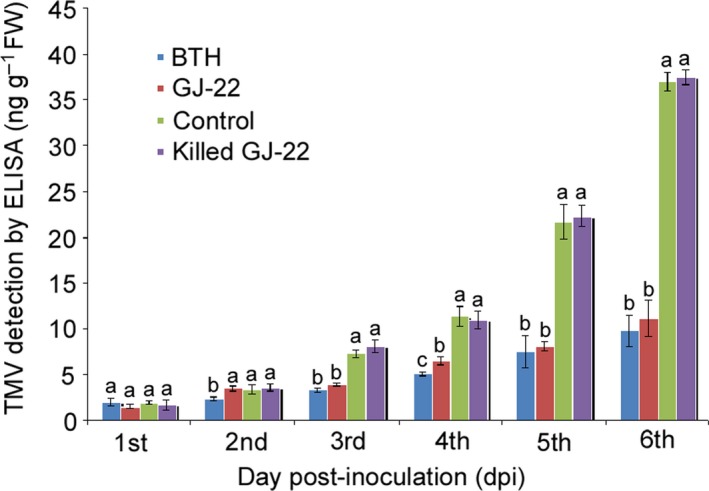
TMV accumulation in inoculated leaves of *N. benthamiana*. The plants were pre‐treated with the indicated treatments before TMV inoculation. The TMV concentration was determined by ELISA. The data are expressed as the mean values with the standard deviations of 12 leaf samples from individual plants. The statistical comparisons are between the different treatments within the same sampling time point. Different letters indicate significant differences using Fisher's LSD (*P* = 0.05).

TMV accumulation in all treatments increased gradually over time, but faster accumulation was detected in the killed GJ‐22 and ultrapure water treatment samples (Fig. 3). On the 6th day post‐inoculation (dpi), the TMV accumulations were 73.5% and 69.8% lower in the BTH and GJ‐22 suspension‐treated leaves, respectively, compared with the water control. The TMV accumulations remained non‐significantly different between the BTH‐ and GJ‐22‐treated leaves at most of the testing time points, suggesting that, similar to BTH‐induced SAR, the pattern of protection against TMV resulted from GJ‐22 inoculation.

#### Defensive enzyme activity assay

**Figure 4:**
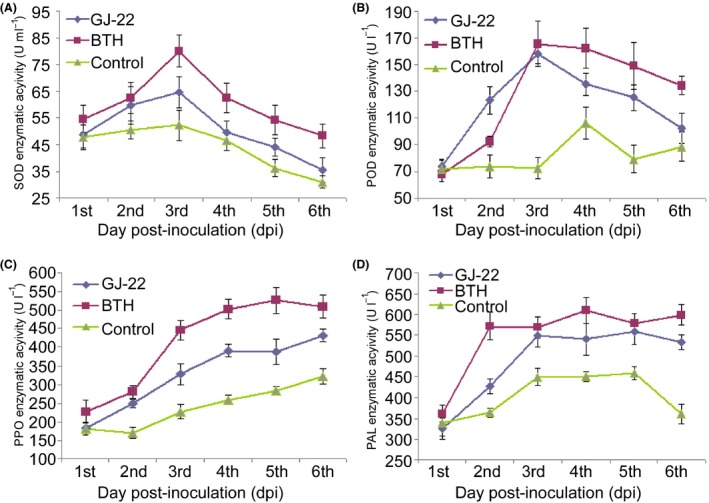
Enhancement of defensive enzyme activity by *R. palustris* GJ‐22. A, B. Activities of the ROS‐scavenging enzymes SOD and POD. C, D. Activities of the pathogenesis‐related defensive enzymes PPO and PAL. The plants were pre‐treated with the indicated treatments before TMV inoculation. Enzyme activity was determined by ELISA. The data are expressed as the mean values with the standard deviations of 12 leaf samples from individual plants.

The comparisons of the enzymatic activities were made between the BTH‐, GJ‐22 suspension‐ and ultrapure water‐treated plants. The enzymatic activities increased more dramatically after TMV inoculation in the BTH‐ and GJ‐22‐treated plants. Superoxide dismutase (SOD) activity reached the maximum level on the 3rd dpi in all treated plants, and 53% and 23% higher activities were observed in the BTH‐ and GJ‐22‐treated plants, respectively, compared with the activities of the ultrapure water‐treated plants on that day (Fig. 4A). The activities then dropped to the minimum on the 6th dpi.

A similar trend was observed for peroxidase (POD) activity. The activity reached the maximum level on the 3rd dpi in the BTH and GJ‐22 suspension‐treated plants but peaked 1 day later in the ultrapure water‐treated plants (Fig. 4B). The maximum POD activities were 49% and 55% higher in the BTH‐ and GJ‐22‐treated plants, respectively, than the ultrapure water‐treated plants. Steadily increasing polyphenol oxidase (PPO) activities over time were observed in all treated plants, but the BTH‐ and GJ‐22‐treated plants achieved higher activities than the ultrapure water‐treated plants at all testing moments (Fig. 4C).

Phenylalanine ammonia‐lyase (PAL) activity reached the maximum level at the 2nd dpi in the BTH‐treated plants and on the 3rd day in the GJ‐22‐ and ultrapure water‐treated plants (Fig. 4D). Unlike the ultrapure water‐treated plants, the BTH‐ and GJ‐22‐treated plants maintained high levels of PAL activity after peaking.

#### Transcription of defence‐related protein genes

**Figure 5:**
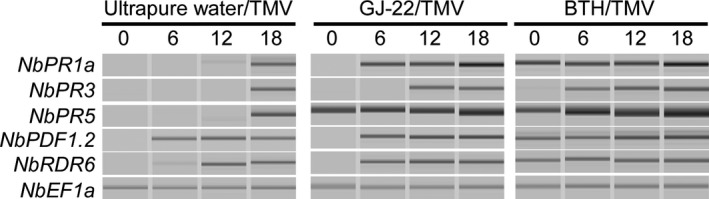
Timings of *NbPR1a*,* NbPR5*,* NbPR3*,* NbPDF1.2* and *NbRDR6* gene transcription in TMV‐inoculated leaves of *N. Benthamiana* that were pre‐treated with the indicated treatments. The leaves were harvested at the indicated time points for extraction of the total RNA. The transcription levels of the genes were semi‐quantified by RT‐PCR using *NbEF‐1α* as an internal reference and then visualized by capillary electrophoresis.

As Fig. 5 illustrates, the transcripts of all of the tested genes with the exception of *NbPDF1.2* appeared detectable earlier in GJ‐22‐ and BTH‐treated plants than the ultrapure water‐treated plants. In contrast to ultrapure water, GJ‐22 advanced the transcript detections of *NbPR3* and *NbRDR6* by 6 h. Specifically, *NbPR1a* and *NbPR5* were detected on the 0th and 6th dpi, respectively, in the GJ‐22‐treated plants, whereas they remained undetected in the ultrapure water‐treated plants until the 18th hour post‐inoculation (hpi). Except for *NbPR5*, the transcriptions of the four other tested genes were detected only after TMV inoculation in the GJ‐22‐treated plants. Except for *NbPR3*, the transcripts of all of the tested genes were detected in the 0th hpi in the BTH‐treated plants.

**Figure 6:**
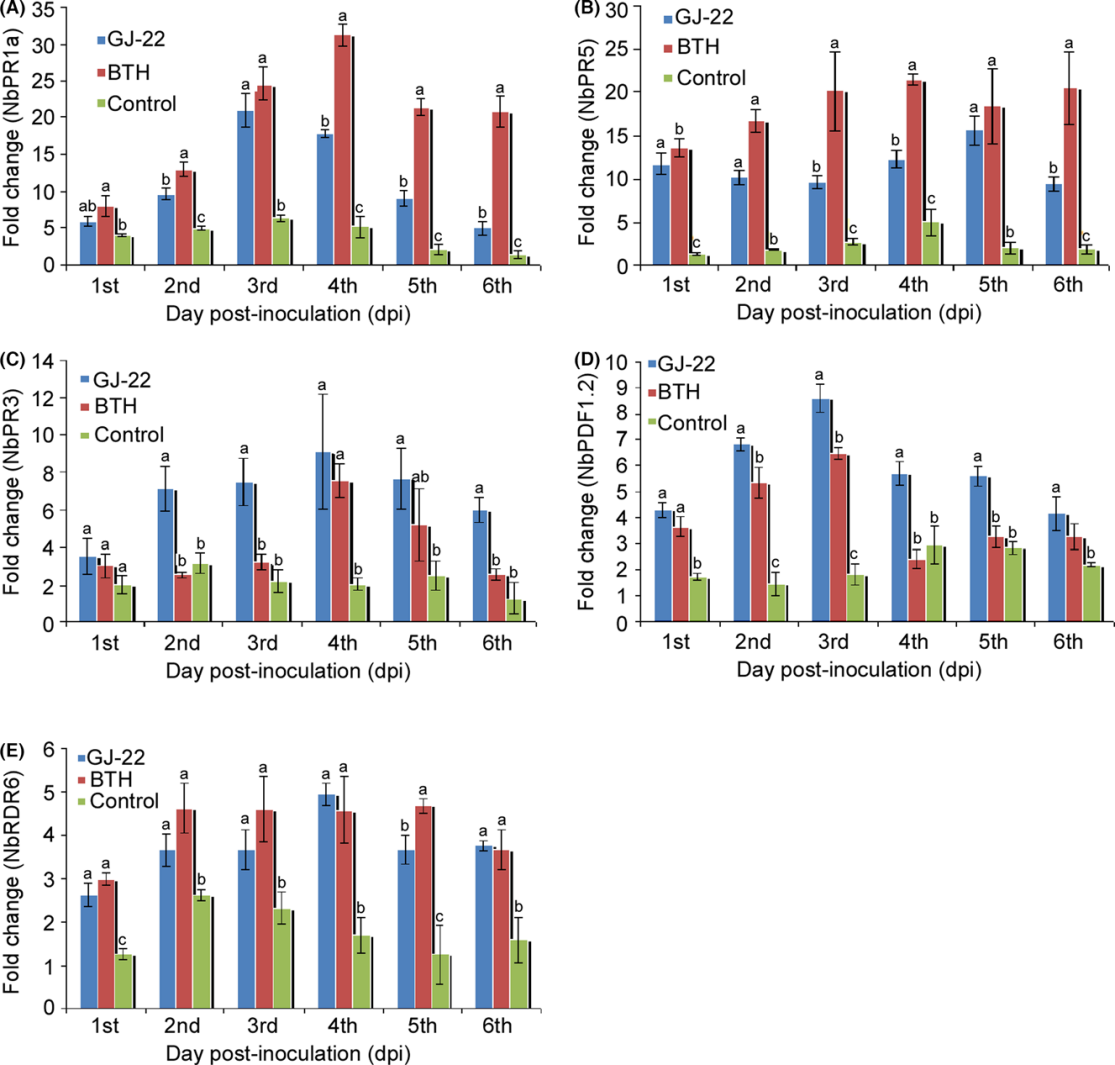
Transcription levels of defence‐related genes in TMV‐inoculated leaves of *N. benthamiana* that was pre‐treated with the indicated treatments. The leaves were harvested at the indicated time points for extraction of the total RNA. A, B. Transcription levels of the *NbPR1a* and *NbPR5* genes, which are associated with the SA‐mediated defence pathway. C, D. Transcription levels of the *NbPR3* and *NbPDF1.2* genes, which are associated with the JA/ET‐mediated defence pathway. E. Transcription levels of the *NbRDR6* gene, which is associated with the RNA‐silencing machinery. The transcription levels of the genes were semi‐quantified by RT‐PCR using *NbEF‐1α* as an internal reference. The data are expressed as the mean values with the standard deviations. The statistical comparisons are between the different treatments within the same sampling time point. The different letters indicate significant differences using Fisher's LSD (*P* = 0.05).

In the GJ‐22‐treated plants, the JA/ET‐responsive genes *NbPR3* (Fig. 6C) and *NbPDF1.2* (Fig. 6D) were upregulated more intensively than in the BTH‐treated plants. The significantly higher transcription levels of these two genes were maintained from the 1st to the 6th dpi in the GJ‐22‐treated plants. Specifically, on the 3rd dpi for *NbPDF1.2* and the 4th dpi for *NbPR3*, 4.7‐ and 4.5‐fold greater upregulations, respectively, were observed compared with the ultrapure water‐treated plants. In contrast, in the BTH‐treated plants, significantly greater upregulations were only observed on the first three dpi for *NbPDF1.2* and on the 4th dpi for *NbPR3*. Conversely, the SA‐responsive genes *NbPR1a* (Fig. 6A) and *NbPR5* (Fig. 6B) were upregulated in a more vigorous manner by BTH than GJ‐22 during the whole time span, although both treatments achieved higher transcription levels of these two genes on the majority of testing days compared with the ultrapure water treatment. For *NbPR1a* in particular, the upregulation began to withdraw on the 4th dpi after peaking on the 3rd dpi in the GJ‐22‐treated plants and decreased to a non‐significant difference relative to the plants treated with ultrapure water. In contrast, this response robustly persisted and remained 15.8‐fold higher on the 6th dpi in the BTH‐treated plants compared with the ultrapure water‐treated plants. A similar pattern contributed by BTH was also observed in the transcription profile of *NbPR5*.

Plant mounts RNA silencing as a basal immune response to stop viruses. In our experiment, *NbRDR6* was upregulated in a quicker and stronger manner in both the GJ‐22‐ and BTH‐treated plants compared with the ultrapure water‐treated plants (Figs 5 and 6E). The maximum differences in the transcription levels between the plants treated with BTH or GJ‐22 and ultrapure water appeared on the 4th and 5th dpi and were 3.7‐fold and 2.9‐fold higher respectively. Notably, only one significant difference was observed between the GJ‐22‐ and BTH‐treated plants. This difference occurred on the 5th dpi and involved the approximate magnitudes of *NbRDR6* upregulation in the two sets of induced plants.

### Field trials

The biological control efficacy of *R. palustris* GJ‐22 against TMV in tobacco was evaluated in a field with severe TMV disease occurrence in Changsha (28°11′ N, 112°58′4 E), Hunan Province, China, in 2012 and 2013. Four treatments, i.e. BTH (chemical control), GJ‐22 suspension (inoculation), autoclaved GJ‐22 suspension (killed control) and ultrapure water (blank control), were set with four replicates by a completely randomized design. The seedlings were sprayed to soaking wet with BTH, GJ‐22 suspension and ultrapure water 7 days after transplantation. For the GJ‐22 suspension treatment, supplementary sprays were conducted once per day for three consecutive days, and the other two treatments were mocked by ultrapure water sprays. For the BTH treatment, two more supplement sprays were conducted after the first spray at an interval of 20 days. The control efficacies of BTH and GJ‐22 remained non‐significantly different in both evaluated years (Table 1). The GJ‐22 significantly increased the yields by 29.8% and 31.7% compared with water control, while BTH only resulted in yield increases of 11.5% and 9.8%. Compared with water control, the increases of first‐class tobacco in 2 years achieved following GJ‐22 inoculation were 27.8% and 39.5%, greater than those following BTH spray, which were 21.8% and 22.6%. There were no significant differences between the killed GJ‐22 inoculation and the water control in terms of disease severity and yield.

**Table 1:** Effect of *R. palustris* GJ‐22 on tobacco disease severity, yield and quality in the field

Treatments	Disease severity (%)		Biocontrol efficacy (%)		Yield (kg/ha)		Yield increase (%)		Increase of first‐class tobacco leaves (%)	
	2012	2013	2012	2013	2012	2013	2012	2013	2012	2013
BTH	12.3 ± 0.7 b	11.4 ± 0.9 b	49.8 ± 4.6 a	55.8 ± 4.9 a	2218.4 ± 91.5 b	2365.8 ± 94.5 b	11.5 ± 1.2 b	9.8 ± 0.6 b	21.8 ± 2.4 b	22.6 ± 2.7 b
GJ‐22	12.1 ± 0.6 b	12.5 ± 0.5 b	50.6 ± 6.7 a	51.6 ± 5.1 a	2582.5 ± 119.8 a	2837.6 ± 97.9 a	29.8 ± 4.9 a	31.7 ± 2.2 a	27.8 ± 2.6 a	39.5 ± 3.7 a
Killed GJ‐22	22.3 ± 0.3 a	23.7 ± 0.4 a	9.0 ± 0.7 b	4.2 ± 0.5 b	2015.5 ± 74.6 c	2173.9 ± 114.9 c	1.3 ± 0.2 c	0.9 ± 0.1 c	3.7 ± 1.4 c	2.5 ± 0.7 c
CK	24.5 ± 0.9 a	25.8 ± 0.7 a	–	–	1989.6 ± 87.9 c	2154.6 ± 97.5 c	–	–	–	–

The mean values from four replicates followed by different letters within a column are significantly different between treatments as determined by Fisher's LSD (*P *=* *0.05). BTH was applied at the concentration of 150 μg ml^−1^. GJ‐22 was applied at the inoculation density of 6 × 10^7^ CFU ml^−1^. Killed GJ‐22 was an autoclaved bacterial suspension with the same density as the GJ‐22 treatment. CK was ultrapure water. The data are expressed as the mean values with the standard deviations.

## Discussion

Viruses cause diseases on all major crops of agronomic importance and cause severe damage in most agriculture areas. Rising concerns regarding food safety and ecological balance make the control of viral diseases particularly challenging. As a strict intracellular pathogen, viral control largely relies on attempts to eradicate insect vectors with the excessive application of pesticides. Additionally, resistance introduced into plants by genetic engineering or conventional breeding can be nullified more easily than that against fungi or bacteria due to the higher genomic plasticity of viruses (Nicaise, 2014). As such, induced resistance conferred by beneficial microorganisms is even more desirable for agricultural practices. Successful exploitation of ISR for the control of viruses, including CMV (cucumber mosaic virus), TMV and ToMoV (tomato mottle virus), has been achieved with many PGPR strains, including *Pseudomonas fluorescens* (Maurhofer *et al*., 1994), *Serratia marcescens* (Raupach *et al*., 1996), *Kluyvera cryocrescens* (Zehnder *et al*., 2000) and *Bacillus* spp. (Murphy *et al*., 2000). Although induced resistances delivered by microorganisms have been considered attractive and alternative approaches for the control of plant diseases, in part due to their minimal conflict with sustainable agriculture practices, the current drawbacks of these approaches are also sternly addressed by scientists. In practice, agro‐ecosystems are filled with variables derived from plant and pathogen genotypes and environmental conditions (Atehnkeng *et al*., 2016; Cray *et al*., 2016). These variables directly influence the outcomes of the disease management desired from biological control agents and thereby complicate efforts to select suitable strains that are able to activate stronger levels of induced resistance or provide maximal protection (Leeman *et al*., 1995; Ton *et al*., 1999). However, accumulating evidence suggests that the microorganisms that are currently under scrutiny in terms of their biological control potential are narrowly dominated by a few genera represented by *Pseudomonas* and *Bacillus* (Choudhary and Johri, 2009; Pieterse, 2009), contradicting the demand for multiple resources of candidate strains with different sets of functional mechanisms that can match the diversity of agro‐ecosystems.

Furthermore, the efficacy of resistance‐inducing treatments in the field remains the key question. For most PGPR strains, establishing the predominant population in soil environments is often difficult (Javaid, 2006). The successful colonization of PGPR requires suitable environmental conditions in terms of soil composition, suitable pH, available water and mineral and carbon sources in addition to the ability to outperform the native microflora in competitive interactions (Persello‐Cartieaux *et al*., 2003). To overcome these constraints, PGPR formulas are often applied to the soil multiple times, and years of repetitive applications can be necessary to acquire an adequate population size (Ferron and Deguine, 2009). Rather than focusing on rhizospheric bacteria, an increasing number of reports indicate that phyllosphere bacterial communities have profound influences on shaping plant biogeographies and ecosystems (Bodenhausen *et al*., 2014). Microorganisms in the phyllosphere are believed to play roles in plant health and growth through similar modes of action with PGPR strains, i.e. growth promotion hormone and antibiotic compound production, resource competition and systemic resistance induction (Berg, 2009). However, the plant protection and growth promotion ability of phyllosphere‐colonizing bacteria is far less studied than that of rhizosphere‐colonizing strains. Recent investigations mainly consist of elucidations of the indigenous bacterial community assemblages in the phyllosphere and their resultant effects on plant traits, whereas the successful introduction of a candidate strain into the phyllosphere, which could positively act on plant growth and defence, has barely been described in the literature (Vorholt, 2012).

As demonstrated in our research, *R. palustris* GJ‐22 successfully colonized the phyllosphere and exhibited a growth promotion effect and a virus resistance‐inducing capability. The efficiencies of both were comparable with those of reported PGPR applications and the chemical inducer BTH when used as a foliar spray. Unlike the rhizosphere, which usually provides a copiotrophic environment for microorganisms, the phyllosphere seems even more forbidding in terms of the survival of introduced bacteria mainly because, in addition to the other microbes and the plant's own defence mechanism, biological agents also need to survive the ultraviolet radiation from sunlight exposure and the oligotrophic conditions of hydrophobic waxy leaf surfaces that prevent plant water evaporation and nutrient leaching. However, this microbe‐unfriendly environment, which makes the establishment of an exogenous bacteria colony intractable, may turn out to be a goldilocks zone for PSB. Unlike PGPR, which depends on soil nutrients, PSB can grow in a photoautotropic mode by harvesting light energy and sequestering carbon from carbon dioxide (Simmons *et al*., 2011). These adaptive traits confer PSB advantages for thriving on the leaf surfaces. This notion was supported by a phyllosphere metagenomic analysis that revealed the presence of a diverse community of anoxygenic phototrophic bacteria (Atamna‐Ismaeel *et al*., 2012). As it reported, individual PSB strains from families, such as *Rhodopseudomonas palustris* and *Rhodobacter sphaeroides*, were delivered onto the phyllosphere and improved the quality and quantity of plant products with remarkable efficiency (Lee *et al*., 2008). Given that some metabolites of PSB can elicit ISR, PSB strains are a rich reservoir to search for phyllosphere‐colonizing biological control agents.

Through our research, an induced resistance of tobacco to TMV by phyllospheric GJ‐22 inoculation was defined by the detection of reduced TMV accumulation, enhanced activities of defensive enzymes and the upregulation of PR genes and the RDR gene. However, the signalling pathway activated in tobacco that was responsible for such induced resistance remained elusive. To answer this question, further efforts need to be made, specifically, efforts involving the use of transgenic plants that are impaired in certain signal transductions. Besides, sufficient evidence in the references indicates the possible overlap between RNA silencing pathways and SA signalling pathways (Campos *et al*., 2014). Therefore, we speculate that the *NbRDR6* upregulation in the BTH‐treated plants was attributable to amplified SA signalling. In contrast, there are currently no data available to associate the RNA silencing mechanism with ISR signalling. Considering the *de facto* enhanced resistance against the virus observed in our experiment, it would be interesting to further investigate the underlying mechanism of GJ‐22‐mediated ISR and its possible correlation with RNA silencing machinery.

In addition, it is also important to mention that the validation of induced resistance simultaneously depends on hormonal signalling and the plant–pathogen system, and in many cases, primed plants could be more susceptible to other type of threats (Derksen *et al*., 2013). This phenomenon largely results from the fact that cross‐talk between signalling pathways can cause suppression or mutual antagonism (Spoel *et al*., 2007). For example, the JA/ET signalling pathway confers resistance to herbivore damage but incurs susceptibility to foliar speck in the tomato due to the suppressed SA signalling pathway (Thaler, 1999). Therefore, to better integrate PSB into conventional agriculture, in addition to isolating more strains with ISR‐eliciting ability, an understanding of the mechanisms responsible for priming and an elucidation of the complex signalling cascades that characterize the signalling mechanisms that influence the ensuing outcomes of host–pathogen interactions are needed. Ultimately, despite their importance, these goals will remain challenging due to the diversity and variety of the plant–pathogen systems as well as the elusiveness and transiency of some of the signalling molecules involved.

## Materials and methods

### Field trials

The seed culture of *R. palustris* GJ‐22 (GenBank Accession Number FJ824030) was incubated in a 250 ml Erlenmeyer flask containing 250 ml of liquid medium, and the flask was manually shaken three to five times per day. A liquid medium containing the following (in g l^−1^) was prepared according to the KOH method with minor modification (Koh and Song, 2007): (NH_4_)_2_SO_4_ 0.1, MgSO_4_ 0.02, Na_2_CO_3_ 0.5, K_2_HPO_4_ 0.05, NaCl 0.02 and yeast extract 0.15 (pH = 6.5–7.0). All cultures were incubated under anaerobic conditions at 30°C and 6500 lux in a light incubator (PRX‐450D, Hangzhou, China) for 7 days. Five 1 L Erlenmeyer flasks were used to prepare the bacterial suspension. The inoculation volume was 5% (v/v) for each flask, and the incubation time was 7 days. Bacterial cells were pelleted from liquid culture by centrifugation (8000 rpm, 15 min) and re‐suspended with ultrapure water. The bacterial suspension was adjusted to the inoculation density of 6 × 10^7^ CFU ml^−1^. The SAR‐inducing Chemical BTH (Syngenta, Research Triangle Park, NC, USA) was used as the chemical control at the concentration of 150 μg ml^−1^ (Friedrich *et al*., 1996).

*Nicotiana tabacum* L. cv. Samsun NN (harbouring the *N* gene) seeds were germinated on Murashige and Skoog medium plates (Thermo Fisher Scientific, USA) in a growth chamber at 25°C in the dark after surface sterilization using 5% sodium hypochlorite for 15 min and five rinses with ultrapure water. After 14 days, the germinated seeds were then individually transferred to culture pots (10 × 10 × 15 cm) containing pH‐balanced peat moss as the base substrate (pH = 5.5–6.5, total N 0.8%; Klasmann‐Deilmann GmbH, Geeste, Germany) in a growth chamber. The culture conditions were set at 25 ± 2°C under a 7500 lux fluorescent light intensity with a cycle of 14‐h/10‐h day/night. After 21 days of cultivation, the tobacco seedlings were evenly transplanted into the experimental plot. Each plot was 100 m^2^ (10 × 10 m) and contained 100 tobacco seedlings.

Apart from treatments, normal agronomic practices were applied in the field without any chemical pesticides. On the 90th day after transplantation, the disease index, quality and yield of the tobacco leaves were recorded. All 100 plants in each plot were investigated for the records. The quality was evaluated as described by Liang *et al*. (2010). The disease index was evaluated based on visual observation and categorized into four levels: 0 = no observed symptoms; 1 = light mottling and a few thin yellow veins; 2 = unevenly distributed mottling and vein clearing on the leaf; 3 = mottling, leaf distortion and stunting; and 4 = severe mottling, leaf curling and stunting. The following equations were used to calculate the disease severity and control efficacy as described by Jiang‐Gang Li and Dong‐Dong Niu (Li *et al*., 2011). Disease severity (%) = [∑ (the number of diseased plants in this index × disease index)/(100 × the highest disease index)] × 100. Control efficacy (%) = [(Disease severity of ultrapure water‐treated – Disease severity of BTH‐ or GJ‐22‐suspension‐treated)/(Disease severity of ultrapure water‐treated)] × 100.

### Plant growth and seed germination under axenic conditions

#### Determination of indole‐3‐acetic acid and 5‐aminolevulinic acid

The GJ‐22 seed culture was inoculated into 250 ml Erlenmeyer flasks containing liquid medium with or without the IAA precursor tryptophan (3 mM) and with or without the ALA precursor glycine and succinate (15 mM). The bacterial supernatants for IAA and ALA quantification were separately sampled from the cultures at the following time points: 0th, 12th, 24th, 36th, 48th, 60th, 72nd, 84th, 96th and 108th hpi. Twelve cultures were sampled at each time point.

The IAA production of GJ‐22 was quantified as described by Wong *et al*. (2014) with minor modification. One hundred microlitres of bacterial supernatant from the anaerobic culture and dilutions of standard IAA were individually mixed with the same volume of Salkowski's reagent (150 ml of concentrated H_2_SO_4_, 7.5 ml of 0.5 M FeCl_3_·6H_2_O, 250 ml of distilled water). The mixture was incubated in a 96 well plate at room temperature for 30 min. The appearance of a pink colour in the mixture indicated the existence of indoles. The plate was then read by a microplate spectrophotometer at 530 nm, and the quantity of IAA was determined according to a standard curve prepared with dilutions of standard IAA.

The ALA production of GJ‐22 was quantified as described by Mauzerall and Granick (1956) with some modifications. Bacterial supernatant (0.5 ml) from the anaerobic culture and dilutions of standard ALA were individually mixed with 0.5 ml of 1M sodium acetate buffer (pH = 7.4) and 50 μl of acetylacetone. The mixtures were placed in a water bath at 100°C for 15 min. After cooling to room temperature, 100 μl of each mixture was individually added into the same volume of modified Ehrlich's reagent (1 g *p*‐dimethylaminobenzaldehyde, 42 ml of glacial acetic acid, 8 ml of 70% (v/v) perchloric acid) pre‐placed in a 96 well plate. The plate was then read by a microplate spectrophotometer at 566 nm, and the quantity of ALA was determined according to a standard curve prepared with dilutions of standard ALA.

#### Phyllospheric colonization of R. palustris GJ‐22

Kanamycin and cycloheximide were used to develop the intrinsically antibiotic‐resistant strain of GJ‐22 (Hameeda *et al*., 2008). Through increasing concentration gradient culture, strain GJ‐22‐1 was obtained and exhibited a combinational resistance level to the two antibiotics (kanamycin at the concentration of 180 μg ml^−1^ and cycloheximide at the concentration of 100 μg ml^−1^). Strain GJ‐22‐1 was subcultured 10 times on antibiotic‐added plates to stabilize the resistance. To verify the hereditary stability of antibiotic resistance of strain GJ‐22‐1, it was cultured on plates without the two antibiotics for 25 generations. The obtained strain GJ‐22‐1 was then cultured on antibiotic‐added plates and exhibited similar growth condition with the original strain GJ‐22‐1. The strain GJ‐22‐1 was later used in phyllospheric colonization test on tobacco.

After spraying with GJ‐22‐1 suspension, 12‐leaf samples from individual tobacco plants were harvested at seven time points, i.e. the 0th, 1st, 3rd, 7th, 15th, 30th, 45th and 60th day post‐inoculation. The leaves collected from each time point were weighed and ground together with ultrapure water. Nine millilitres of ultrapure water for each gram of leaf was used. Ten grams of mixture containing one gram of leaf tissue from each time point was centrifuged to obtain the cell suspension. Serial dilutions of the cell suspension were daubed on a medium plate supplemented with kanamycin and cycloheximide and incubated under the condition described in the enrichment and isolation section. The number of UFCs per gram of fresh leaf was determined after 7 days of incubation.

#### Seed germination test

*Nicotiana benthamiana* (susceptible to TMV infection) seeds were surface‐sterilized and rinsed, and GJ‐22 suspension was prepared as described in the field trials section. The seeds were then placed on a filter paper that was sprayed to soaking wet with GJ‐22 suspension, autoclaved GJ‐22 suspension (killed control) or ultrapure water in a Petri dish. One hundred seeds were evenly laid on each filter paper, and the Petri dishes were placed in a growth chamber at 25°C in the dark. The germination of the tobacco seeds was counted 7 days after the treatments.

#### Plant growth test

*Nicotiana benthamiana* (susceptible to TMV infection) seedlings were prepared and transferred to culture pots as described in the field experiment section. After 14 days of cultivation, seedlings with the same growth condition were selected and divided into nine groups for the different treatments, and 100 plants were used for each treatment. These treatments were the following: (i) ultrapure water as the blank control; (ii) GJ‐22 suspension as the inoculation group; (iii) autoclaved GJ‐22 suspension as the killed control; (iv) GJ‐22 suspension + tryptophan; (v) autoclaved GJ‐22 suspension + tryptophan; (vi) GJ‐22 suspension + glycine and succinate; (vii) autoclaved GJ‐22 suspension + glycine and succinate; (viii) tryptophan; and (ix) glycine and succinate. Tryptophan was added to the suspensions at the final concentration of 3 mM as a precursor of IAA. Glycine and succinate were added to the suspensions at the final concentrations of 15 mM as the precursors of ALA. All treatments were applied to the tobacco leaves via a foliar spray to soaking wet. The root and shoot lengths of the plants were measured 21 days after the treatments, and the dry mass of each whole plant was then weighed after drying at 80°C for 7 days.

### Post‐challenge immune response assays

#### Plants and pathogen

*Nicotiana benthamiana* (susceptible to TMV infection) seedlings were prepared with the same method described in the field experiment section. After 14 days of cultivation, healthy seedlings with the same growth condition were selected for the treatments. The TMV U1 strain was propagated and purified from its systemic host *Nicotiana tabacum* cv. Huangmiaoyu as described by Pin Su (Su *et al*., 2015).

#### Treatments and TMV inoculation

Three treatments, i.e. BTH, GJ‐22 suspension and autoclaved GJ‐22 suspension, were set at the concentrations as described in the field experiment part, and ultrapure water was set as the blank control. All treatments were applied to the *N. benthamiana* leaves via foliar spray until the leaves were soaking wet. After 7 days of treatment, the tobacco seedlings were subjected to TMV inoculation. The purified TMV particles were diluted to 1 μg ml^−1^ with the PB solution (pH = 7, 0.2 M), and the third leaf of each plant of the different treatments was inoculated with 20 μl of TMV solution by rubbing in the presence of carborundum.

#### TMV accumulation and defensive enzyme activity assay

Enzyme‐linked immunosorbent assay (ELISA) was conducted according to the manufacturer's instructions (IBL‐America, Minneapolis, Minnesota, USA). Leaves inoculated with TMV particles from the pre‐treated plants as described above were collected at the time points of the 1st, 2nd, 3rd, 4th, 5th and 6th day post‐inoculation. Twelve 1 gram fresh leaf tissue samples from each treatment from the different time points were collected from the inoculated leaf. Each gram of fresh leaf tissue was flash‐frozen and ground in 1 ml of PBS buffer (pH = 7.4). The ground tissues were centrifuged (8500 rpm, 20 min), and the supernatants were subjected to ELISA. The TMV accumulation and defensive enzyme activities of PAL, PPO, POD and SOD were determined with a microplate spectrophotometer at 450 nm.

#### RNA extraction and RT‐PCR analysis

Leaves inoculated with TMV particles were collected for total RNA extraction from the pre‐treated plants at the time points of the 0th, 6th, 12th and 18th hours of post‐inoculation and the 2nd, 3rd, 4th, 5th and 6th dpi. Four 0.5 g fresh leaf tissue samples from each treatment at the different time points were collected from the inoculated leaf and then flash‐frozen in liquid nitrogen for storage at −80°C. Total RNA was extracted using the MiniBEST plant RNA extraction kit (TaKaRa Biotechnology, Dalian, China). The cDNA was synthesized using PrimeScript™ 1st Strand cDNA synthesis kits (TaKaRa Biotechnology, Dalian, China). The transcript levels of two SA‐responsive genes, i.e. *NbPR1a* and *NbPR5*, two JA/ET‐responsive genes, i.e. *NbPR3* and *NbPDF1.2*, and one host RNA‐dependent RNA polymerase gene, i.e. *NbRDR6,* were semi‐quantified using TransStart^®^ Green qPCR SuperMix UDG (TransGen Biotech, Beijing, China) and a PTC‐200 Real‐Time PCR system (Bio‐Rad, Hercules, California, USA). The specific primers for genes were as follows: *NbPR1a*‐F: AGGGATCCATGGGATTTGTTCTCTTT, R: AGGAGCTCTTAGTATGGACTTTCGCC (Lee *et al*., 2016); *NbPR3*‐F: AAAGGGATTCTACAGTTAC, R: AGGATTGTTTAGCAGGT (Zhu *et al*., 2014); *NbPR5*‐F: TGAGGAGGATGAATAGA, R: AAAGCCTAACAAGTGC (Zhu *et al*., 2014); *NbPDF1.2*‐F: AACTTGTGAGTCCCAGAG, R: GGATACCTTTCTACCACC (Zhu *et al*., 2014); and *NbRDR6*‐F: CTTTGGATGAGAAGTGCCTA, R: TTTGGGACAAGCTCAAGTC (Qin *et al*., 2012). *NbEF‐1α* was selected for an internal reference gene, and the following primers were used: *NbEF‐1α*‐F: TGCCTTGTGGAAGTTTGAGACC, R: GGTGGAGTCAATAATCAGGACAGC (Qin *et al*., 2012). The primers at the final concentrations of 0.2 μM each and 0.8 μl of cDNA templates were added to 20 μl of total reaction system. The PCR cycling conditions comprised an initial polymerase activation step at 90°C for 5 min followed by 35 cycles of 95°C for 15 s, 60°C for 50 s and 72°C for 1 min. The PCR efficiency for each gene was checked according to the slope of the standard curve generated from the amplification. The target gene expression levels are conveyed as the fold changes in the transcript levels compared with the reference gene using the comparative $2-\Delta \Delta^{C_{T}}$method. The mean deviation was calculated from the standard deviation (SD) in the $\Delta \Delta C_{T}$ ▵▵*C*_T_ value using the formula $2-\Delta \Delta^{C_{T}+\mathrm{SD}}$. All reactions were performed in triplicate.

The RT‐PCR products of leaf tissues sampled within 24 h post‐inoculation were analysed using capillary electrophoresis (QIAxcel; QIAGEN, Germantown, Maryland, USA) to visualize the timing of the upregulation of the defence genes. The target gene expression levels 24 h post‐inoculation are presented and compared in bar diagrams.

### Statistical analysis

All of the data collected in this research were from four experimental repeats. The data are presented as the mean ± the standard deviation, and significant differences between the treatments and the controls were determined with analyses of variance using spss Statistics 17.0 software (IBM Corp., New York, USA).

## Author contributions

YL, PS, XGZ, DYZ, XQT and SBZ designed the experiments and analysed the data. PS, CGL, JP and ZZ performed the experiments. XYL, JEC, QPY and JP contributed reagents/materials. PS wrote the manuscript.

## Conflict of interest

The authors declare no competing financial interests.
